# Virgin Coconut Oil Supplementation Prevents Airway Hyperreactivity of Guinea Pigs with Chronic Allergic Lung Inflammation by Antioxidant Mechanism

**DOI:** 10.1155/2020/5148503

**Published:** 2020-01-27

**Authors:** Luiz Henrique C. Vasconcelos, Maria da Conceição C. Silva, Alana C. Costa, Giuliana A. de Oliveira, Iara Leão Luna de Souza, Renato F. Righetti, Fernando R. Queiroga, Glêbia A. Cardoso, Alexandre S. Silva, Patrícia M. da Silva, Giciane C. Vieira, Iolanda de F. L. C. Tibério, Marta S. Madruga, Fabiana de A. Cavalcante, Bagnólia A. da Silva

**Affiliations:** ^1^Programa de Pós-graduação em Produtos Naturais e Sintéticos Bioativos, Centro de Ciências da Saúde, Universidade Federal da Paraíba, João Pessoa, PB, Brazil; ^2^Departamento de Fisiologia e Patologia, Centro de Ciências da Saúde, Universidade Federal da Paraíba, João Pessoa, PB, Brazil; ^3^Graduação em Farmácia, Departamento de Ciências Farmacêuticas, Centro de Ciências da Saúde, Universidade Federal da Paraíba, João Pessoa, PB, Brazil; ^4^Departamento de Ciências Biológicas e Saúde, Universidade Estadual de Roraima, Boa Vista, RR, Brazil; ^5^Faculdade de Medicina FMUSP, Universidade de São Paulo, São Paulo, SP, Brazil; ^6^Hospital Sírio-Libanês, Serviço de Reabilitação, São Paulo, SP, Brazil; ^7^Departamento de Biologia Molecular, Centro de Ciências da Saúde, Universidade Federal da Paraíba, João Pessoa, PB, Brazil; ^8^Departamento de Educação Física, Centro de Ciências da Saúde, Universidade Federal da Paraíba, João Pessoa, PB, Brazil; ^9^Departamento de Morfologia, Centro de Ciências da Saúde, Universidade Federal da Paraíba, João Pessoa, PB, Brazil; ^10^Departamento de Engenharia de Alimentos, Centro de Tecnologia, Universidade Federal da Paraíba, João Pessoa, PB, Brazil; ^11^Departamento de Ciências Farmacêuticas, Centro de Ciências da Saúde, Universidade Federal da Paraíba, João Pessoa, PB, Brazil

## Abstract

Asthma is a chronic inflammatory disease of the airways characterized by immune cell infiltrates, bronchial hyperresponsiveness, and declining lung function. Thus, the possible effects of virgin coconut oil on a chronic allergic lung inflammation model were evaluated. Morphology of lung and airway tissue exhibited peribronchial inflammatory infiltrate, epithelial hyperplasia, and smooth muscle thickening in guinea pigs submitted to ovalbumin sensitization, which were prevented by virgin coconut oil supplementation. Additionally, in animals with lung inflammation, trachea contracted in response to ovalbumin administration, showed a greater contractile response to carbachol (CCh) and histamine, and these responses were prevented by the virgin coconut oil supplementation. Apocynin, a NADPH oxidase inhibitor, did not reduce the potency of CCh, whereas tempol, a superoxide dismutase mimetic, reduced potency only in nonsensitized animals. Catalase reduced the CCh potency in nonsensitized animals and animals sensitized and treated with coconut oil, indicating the participation of superoxide anion and hydrogen peroxide in the hypercontractility, which was prevented by virgin coconut oil. In the presence of L-NAME, a nitric oxide synthase (NOS) inhibitor, the CCh curve remained unchanged in nonsensitized animals but had increased efficacy and potency in sensitized animals, indicating an inhibition of endothelial NOS but ineffective in inhibiting inducible NOS. In animals sensitized and treated with coconut oil, the CCh curve was not altered, indicating a reduction in the release of NO by inducible NOS. These data were confirmed by peribronchiolar expression analysis of iNOS. The antioxidant capacity was reduced in the lungs of animals with chronic allergic lung inflammation, which was reversed by the coconut oil, and confirmed by analysis of peribronchiolar 8-iso-PGF2*α* content. Therefore, the virgin coconut oil supplementation reverses peribronchial inflammatory infiltrate, epithelial hyperplasia, smooth muscle thickening, and hypercontractility through oxidative stress and its interactions with the NO pathway.

## 1. Introduction

Functional foods have properties in the body, with respect to the metabolic and physiological role, which may or may not have health properties, that is, a beneficial relation between food and a certain health condition [1]. Several foods are classified within this classification, highlighting the coconut oil, which is recognized as a food for supplementation, based on safety and efficacy data [2].

The coconut oil (species: *Cocos nucifera* L., family: Arecaceae) is a product obtained from the mature seed of coconut or copra (dry coconut pulp), which is mainly used to obtain the oil, being constituted between 65 and 75% of it [3], widely used in food and industry [4, 5].

This oil is rich in medium-chain saturated fatty acids, effective against the development of cardiovascular and inflammatory diseases [6], as well as antioxidant compounds (carotenoids and tocopherols) and vitamins [7]. In addition, it is described in the literature that virgin coconut oil has a composition of unsaponifiable compounds—mostly polyphenols and tocotrienols with antioxidant activity—superior to oils obtained by conventional methods such as cooling or enzymatically [4].

Among the pharmacological properties described for this oil are the anti-inflammatory [8], antihypertensive [9] to prevent coronary disease [10], and cardioprotective [11]. Thus, because of its actions on the inflammatory process, coconut oil is a potential candidate in the adjuvant therapy of several chronic inflammatory diseases, such as allergic asthma.

Asthma is a chronic inflammatory disease of the airways in which many innate and adaptive cells of the immune system act in conjunction with epithelial cells to promote bronchial hyperactivity, characterized as the tendency of smooth muscle cells to react exacerbatedly to nonspecific stimuli, such as cold air and exercise, in addition to excess production of mucus, remodeling of the airway wall and narrowing of the lumen of these conductive pathways. In susceptible patients, it leads to dyspnea and repeated periods of shortness of breath, wheezing during breathing, and chest tightness [12].

Despite the great diversity of drugs for the treatment of this disease, this is still done in a palliative and/or preventive way; therefore, new therapeutic approaches are necessary with the purpose of limiting or at least make acute crises less frequent or that potentiate the effects of drugs currently available for the treatment of asthma and, then, reducing the development of acute attacks.

In view of the above, virgin coconut oil presents potential as a functional food with health properties, emerging as a complementary therapy to prevent or reduce asthmatic crises. Therefore, the aim of this study was to evaluate a possible modulating activity of virgin coconut oil on the parameters of airway smooth muscle contraction, pulmonary inflammation, and oxidative stress, in order to characterize its effects on the pathophysiological process of chronic allergic lung inflammation.

## 2. Materials and Methods

### 2.1. Animals

Male and female adult guinea pigs (*Cavia porcellus*), approximately 300-350 g, were obtained from the Biotherium of Research Institute in Pharmaceuticals and Medicine (IPeFarM/UFPB). The animals were maintained under controlled ventilation and temperature (21 ± 1°C) with water and food (Presence®) *ad libitum* in a 12 h light-dark cycle (lights on from 6 a.m to 18 p.m). The experimental procedures were performed following the principles of guidelines for the ethical use of animals in applied etiology studies [13] and from the Brazilian Guide for the Production, Maintenance or Use of Animals in Teaching or Scientific Research Activities, from Conselho Nacional de Controle de Experimentação Animal (CONCEA) [14] and were previously approved by the Ethics Committee on Animal Use of UFPB (protocol no 0410/13).

### 2.2. Drugs

Calcium chloride (CaCl_2_), magnesium sulphate (MgSO_4_), sodium chloride (NaCl), and potassium chloride (KCl) were purchased from Vetec Química Fina Ltda. (Brazil). Sodium bicarbonate (NaHCO_3_) and glucose (C_6_H_12_O_6_) were purchased from Dinâmica (Brazil). Potassium monobasic phosphate (KH_2_PO_4_), sodium hydroxide (NaOH), and hydrochloric acid (HCl) were purchased from Nuclear (Brazil). These substances, except glucose, NaHCO_3_, and NaCl, were diluted in distilled water to obtain each solution, which were maintained under refrigeration.

Catalase was purchased from Cayman Chemical (Brazil), and saline (0.9% NaCl) was purchased from Fresenius Kabi LTDA (Brazil). Carbamylcholine hydrochloride (CCh), histamine dihydrochloride, iberiotoxin (IbTX), isoproterenol, Cremophor®, tetramethoxypropane, perchloric acid, Mayer's hematoxylin, eosin, 1,1-diphenyl-2-picrylhydrazyl (DPPH), apocynin, arachidonic acid (AA), ethylenediaminetetraacetic acid (EDTA), formaldehyde, nifedipine, ovalbumin (OVA) (grade V), and tempol were acquired from Sigma-Aldrich (Brazil). All substances were diluted in distilled water as needed for each experimental protocol. The carbogen mixture (95% O_2_ and 5% CO_2_) was obtained from White Martins (Brazil).

### 2.3. Experimental Groups

Animals were randomly divided into six groups (5-8 animals/group): guinea pigs submitted to nebulization with saline solution (Ctrl); guinea pigs with chronic allergic pulmonary inflammation, submitted to nebulization with OVA (Asth); guinea pigs with chronic allergic lung inflammation treated with dexamethasone (2 mg/kg/day) (Asth+Dexa); guinea pigs with chronic allergic lung inflammation supplemented with virgin coconut oil (1 g/kg/day) (Asth+VCO1); guinea pigs with chronic allergic lung inflammation supplemented with virgin coconut oil (2 g/kg/day) (Asth+VCO2); and guinea pigs with chronic allergic lung inflammation supplemented with virgin coconut oil (4 g/kg/day) (Asth+VCO4).

### 2.4. Virgin Coconut Oil

The virgin coconut oil was purchased from a natural product store in the municipality of João Pessoa/PB, Brazil, brand Dr. Orgânica® (lot no. V15 245), extracted by cold pressing, not refined. A sample of the oil (500 mL) was used in order to determine the acidity, peroxide and saponification indexes, and the fatty acid profile.

### 2.5. Determination of Fatty Acid Composition of the Virgin Coconut Oil

Methyl esters of the oil sample were obtained by esterification. Identification and quantification of the fatty acid esters were performed by gas chromatography (GC), using helium as the entrainment gas (flow rate of 1 mL/min).

The GC conditions were injector temperature of 250°C, initial furnace temperature of 100°C, increasing at 2.5°C/min to 240°C, remaining for 40 min, with a total run time of 96 min. The detector temperature was 250°C, aux gas flow: helium 25 mL/min, hydrogen 30 mL/min, and synthetic air 300 mL/min. 1.0 *μ*L aliquots of the esterified sample were injected into the injector, and the chromatograms were recorded. Fatty acids were identified by comparing the retention times of the methyl esters of the samples with Supelco ME19-Kit standards (Fatty Acid Methyl Esters C4-C24). The analyses were performed in duplicate.

### 2.6. Determination of the Acid Index of the Virgin Coconut Oil

Virgin coconut oil samples, completely liquid, were homogenized. Next, 2 g of the sample was added in an Erlenmeyer flask and filled with 25 mL of neutral ether-alcohol solution (2 : 1). Two drops of acid/basic phenolphthalein indicator were added, and titration was carried out with 0.1 M sodium hydroxide solution until the appearance of pink staining, which should persist for 30 sec.

The absolute acidity index (KOH/g oil) was calculated by the formula: (*v* × *f* × 5.61)/*P*, where *v* is the volume (mL) of NaOH solution spent in the titration of the sample, *f* the NaOH solution factor, and *P* the mass (g) of the sample [15]. The analyses were performed in triplicate.

### 2.7. Determination of the Peroxide Index of the Virgin Coconut Oil

Acetic acid-chloroform solution (3 : 2) (30 mL) was added to 5 g of the virgin coconut oil in an Erlenmeyer flask under gentle stirring, followed by the addition of 0.5 mL of saturated iodide solution of potassium. Subsequently, the mixture was left out in the light for 1 minute, and then, 30 mL of distilled water were added. This content was titrated with 0.1 N sodium thiosulphate solution under constant stirring until the yellow color disappeared. Then, 0.5 mL of 1% starch solution (indicator) was added, continuing the titration until the complete disappearance of the blue color.

The peroxide index (meq/kg sample) was calculated by the formula: [(*A* − *B*) × *N* × *f* × 1000]/*P*, wherein *A* is the volume (mL) of sodium thiosulfate spent on titration of the sample and *B* on the titration of the blank, *N* is the normality of the sodium thiosulphate solution, *f* is the factor of sodium thiosulphate solution, and *P* is the mass (g) of the sample. The analyses were performed in triplicate.

### 2.8. Determination of the Saponification Index of the Virgin Coconut Oil

The coconut oil sample was melted and filtered on filter paper to remove impurities and moisture traces. Then, 5 mL of the sample was added 50 mL of alcoholic KOH solution. A condenser was connected and allowed to boil gently until complete saponification of the sample, and then, its cooling was done. Then, 1 g of the phenolphthalein indicator was added, and the solution was titrated with 0.5 M HCl until the complete disappearance of the pink color.

The saponification index (mg KOH/g oil) was calculated by the formula: [26.06 × *f* × (*B* − *A*)]/*P*, where *A* is the volume (mL) of HCl spent on the titration of the sample, *B* is the volume spent on the blank titration, *f* is the 0.5 M HCl solution factor, and *P* is the mass (g) of the sample. The analyses were performed in triplicate.

### 2.9. Induction of Chronic Allergic Lung Inflammation

Animals were individually placed in a closed polyacrylic box coupled to an ultrasonic nebulizer for nebulization. Then, guinea pigs were nebulized with OVA in saline solution for a maximum of 15 min or until the onset of sneezing, coryza, coughing, and/or drawing of the thoracic wall, characterizing the respiratory distress; the Ctrl received only saline solution (NaCl 0.9%). The time that the guinea pigs remained in nebulization was defined as the inhalation time.

The protocol consisted of seven inhalations performed in four weeks, with 96 h intervals between each inhalation, with OVA concentration being increased (1-5 mg/mL) to avoid tolerance. In the first four inhalations, the guinea pigs were submitted to OVA 1 mg/mL; in the fifth and sixth inhalations, the animals received OVA 2.5 mg/mL; and at the seventh inhalation, they received OVA 5 mg/mL. After 72 h of the last inhalation, the animals were euthanized by cervical dislocation followed by sectioning of cervical vessels for experimentation. The ctrl was submitted to the same inhalation procedure but only receiving saline solution (adapted from Tibério et al. [16], Angeli et al. [17], Pigati et al. [18], Vasconcelos et al. [19]).

**Figure 1:**
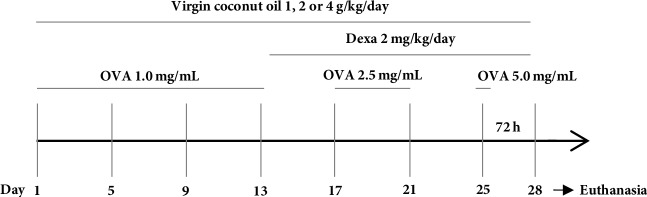
Protocol for induction of chronic allergic pulmonary inflammation in guinea pigs. OVA = ovalbumin; Dexa = dexamethasone.

The groups supplemented with virgin coconut oil received orally, daily doses of 1, 2 or 4 g/kg [9, 20–22], ending the day before the euthanasia of the animal. Guinea pigs from the Asth+Dexa group were treated with the drug at 2 mg/kg/day intraperitoneally, starting 24 hours after the fourth nebulization, to ensure that the animals were sensitized (Figure 1). During this period, dexamethasone treatment was done 5 hours prior to exposure to the antigen [23].

### 2.10. Evaluation of Lung and Bronchial Morphology

Guinea pigs were euthanized by guillotine, the lung and the right extrapulmonary bronchus were isolated, fixed in 10% formaldehyde solution for 24 h, and subjected to standard histological procedures as follows: (1) dehydration, by increasing alcohol series of 70% for 24 h and 80, 96, and 100% (third bath) for 1 h each; (2) diaphanization, by bath in 100% xylene alcohol (1 : 1) for 1 h, followed by two baths in pure xylene for 1 h each; and (3) embedding in paraffin by passing the sample through two baths of liquid paraffin (heated to 50°C) for 1 h each. Next, the samples were embedded in new paraffin. The blocks obtained were cut to 5 *μ*m thick and stained with Mayer's hematoxylin/eosin. For a panoramic analysis of the histological section, the slides were analyzed in the increase lens (I.L.) of ×40 (1500 *μ*m) and ×100 (600 *μ*m).

For bronchial analysis, the evaluated aspects were wall structure, tissue integrity, and cellular migration. In these experiments, 5 slides of 5 different animals were analysed for each group. The photomicrographs of the slides were made with a camera coupled to an optical microscope. The images were calibrated through the Motic Plus program according to the objectives used: 4x and 10x. The histological analysis of the slides was performed by a trained operator who qualitatively analyzed the histological parameters and then quantified statistically by score (below).

1. Preserved histoarchitecture of the airways with the absence of perivascular and peribronchiolar cellular infiltrate
2. Mild degree: an increase of less than 25% in relation to the control
3. Moderate degree: an increase of 25 to 49% in relation to the control
4. Accentuated degree: an increase of 50 to 75% in relation to the control
5. Very pronounced degree: an increase of more than 75% in relation to the control.

### 2.11. Trachea Preparation

Guinea pigs were euthanized by guillotine. The trachea was removed, cleaned of fat and connective tissue, immersed in Krebs solutions, and bubbled with carbogen mixture (95% O_2_ and 5% CO_2_). The Krebs solution composition was (mM): NaCl (118.0), KCl (4.55), MgSO_4_ (5.7), KH_2_PO_4_ (1.1), CaCl_2_ (2.52), NaHCO_3_ (25.0), and glucose (11.0), with pH adjusted to 7.4. To register isometric contractions, tracheal rings (2-3 cm) were suspended in steel rods in organ baths (6 mL), connected to a force transducer (TIM 05), attached to an amplifier (AECAD04F), and connected to an A/D converter into a PC running AQCAD® software (São Paulo, Brazil). The system contained a thermostatic pump model BT 60 that controlled the organ bath's temperature. The trachea resting time was 60 min in a preload tension of 1 g (baseline). During the organ-resting phase, the solution was changed every 15 min to avoid metabolite accumulation [24].

After the stabilization period, the tracheal segments were contracted with CCh 1 *μ*M, and the isometric tension was recorded. The arachidonic acid 0.1 mM was added to the organ bath, when a stable contraction was attained, in order to confirm the presence of epithelium by the presence of arachidonic acid-induced relaxation equal or higher than 50% of maximal tension. In some tracheal rings, the luminal surface was gently rubbed with Krebs wet cotton to remove the epithelial layer. The absence of epithelium was confirmed when arachidonic acid-induced relaxation was absent or lower than 10% of maximal tension [24].

### 2.12. Reactivity Measurement

The trachea was assembled as described previously. After the stabilization period, when the baseline remained constant, the intact epithelium tracheal rings were stimulated with OVA 10 *μ*g/mL [25–28], and the contraction amplitude was compared between all groups.

In another experiments, after the evaluation of epithelium integrity, a cumulative concentration-response curve was obtained to CCh (10 nM to 0.1 mM), an agonist of muscarinic receptors [29], or histamine (10 nM to 3 mM), an agonist of histaminergic receptors [29]. The contractile reactivity was evaluated based on the values of the maximum effect (*E*_max_) and the negative logarithm of the molar concentration of a substance that produced 50% of its maximal effect (pEC_50_) of the contractile agent, calculated from the concentration-response curves obtained. The maximum amplitude obtained from the control concentration-response curve was elected as 100% of contraction, and the other percentages of contraction were calculated related to this value.

### 2.13. Role of ROS and NO Pathways in the Reactivity Measurement

The trachea was assembled as described previously. After checking for the presence of epithelium, the curves to CCh were obtained either in the absence or presence of apocynin 10 *μ*M, an inhibitor of NADPH oxidase [30], and tempol 1 mM, a superoxide dismutase mimetic [31], after 30 min of incubation; or catalase 100 IU/mL, after 10 min incubation for ROS pathway investigation. In other experiments, the curves to CCh were obtained either in the absence or presence of L-NAME 10^−4^ M, an inhibitor of NOS [31], after 45 min incubation for NO pathway investigation.

### 2.14. Antioxidant Activity Assay

In order to obtain the plasma, after euthanasia of the animals, 10 mL of blood was collected through cervical vessel sections, placed immediately in anticoagulant (EDTA) containing test tubes and centrifuged at 1,198 × g for 10 min. The supernatant was then transferred to Eppendorf® tubes and refrigerated at 20°C until analysis [32, 33]. Additionally, to obtain the lung tissue homogenate, the lungs were isolated and frozen at 20°C until preparation of the homogenate. For this, the tissue was weighed, macerated, and homogenized with 10% KCl (1 : 1). Then, the samples were centrifuged (1,198 × g/10 min), and the supernatant was separated for further analysis.

For total antioxidant capacity analysis, an aliquot of 1.25 mg of DPPH was diluted in ethanol (100 mL), kept under refrigeration, and protected from light. In proper centrifuge tubes, 3.9 mL of DPPH solution was added with 100 *μ*L of the samples. These tubes were vortexed and left to stand during 30 min, centrifuged at 7,489 × g at 20°C during 15 min. Then, the samples were read in a spectrophotometer at a wavelength of 515 nm (Biospectro, SP-220 model, Brazil). Results were expressed as percentage of the antioxidant capacity: AOA = 100 − ((DPPH · R)S/(DPPH · R)W · 100), where (DPPH · R)S and (DPPH · R)W corresponding to the concentration of DPPH • remaining after 30 min, measured in the sample (S) and white (W) prepared with distilled water.

### 2.15. Immunohistochemistry for Peribronchiolar iNOS and 8-Iso-PGF2*α* Measurement

The slices obtained as described previously were deparaffinized and rehydrated for immunohistochemistry, treated with Proteinase K (20 min at 37°C followed by 20 min at room temperature), and washed with PBS. Blocking of endogenous peroxidases was performed by incubation with 3% hydrogen peroxide (H_2_O_2_) 10 V (3 × 10 min), and sections of experimental and control (positive and negative) tissue slides were incubated overnight with the anti-iNOS, dilution 1 : 1600 (IS-20 Goat Polyclonal; Oxford Biomed, Resear, MI, USA) or anti-8-iso-PGF2*α*, dilution 1 : 1500 (N-32020 Mouse Monoclonal; BD transduction Lab., CA, USA). The following day, the slides were washed in PBS and incubated with a secondary antibody using ABCKit by Vectastain (Vector Elite-PK-6105 anti-goat) or PK-6102 (anti-mouse). For visualization of positive cells, the slides were washed in PBS, and proteins were visualized using 3,3′-diaminobenzidine chromosome (DAB) (Sigma Chemical Co., St. Louis, MO, USA). Slide sections were contrasted with Harris hematoxylin (Merck, Darmstadt, Germany) and assembled using Entellan microscopy resin (Merck).

The optical density was used to evaluate the expression of iNOS and isoprostane PGF2*α*. Images were captured using a Leica DM2500 microscope (Leica Microsystems, Wetzlar, Germany) and a digital camera (Leica DFC420 Leica Microsystems, Wetzlar, Germany). The images were acquired and processed using Optimas v.4.10 software. We analyzed 10 fields per lamina and one lamina per animal. The images were analyzed using Image-Proplus 4.5 software (NIH, MD, USA). This software allowed a thresholding of the color shades to be developed. These shades represent the positive areas quantified in the previously determined area. The volume fractions of these markers are expressed as percentages of the area.

### 2.16. Statistical Analysis

Data were expressed as the mean and standard error of the mean (S.E.M.), the normality of the variance was verified by the Shapiro Wilk test, and results were statistically analyzed using Student's *t*-test to intergroup comparison or analysis of variance (ANOVA) one-way, followed by Tukey's post-test, for multiple comparisons between experimental groups. Cumulative concentration-response curves were fitted, and pEC_50_ values were obtained by nonlinear regression [34]. Values were significantly different when *p* < 0.05. All data were analyzed by GraphPad Prism® version 5.01 (GraphPad Software Inc., USA).

## 3. Results

### 3.1. Evaluation of Pulmonary and Bronchial Morphology

**Figure 2:**
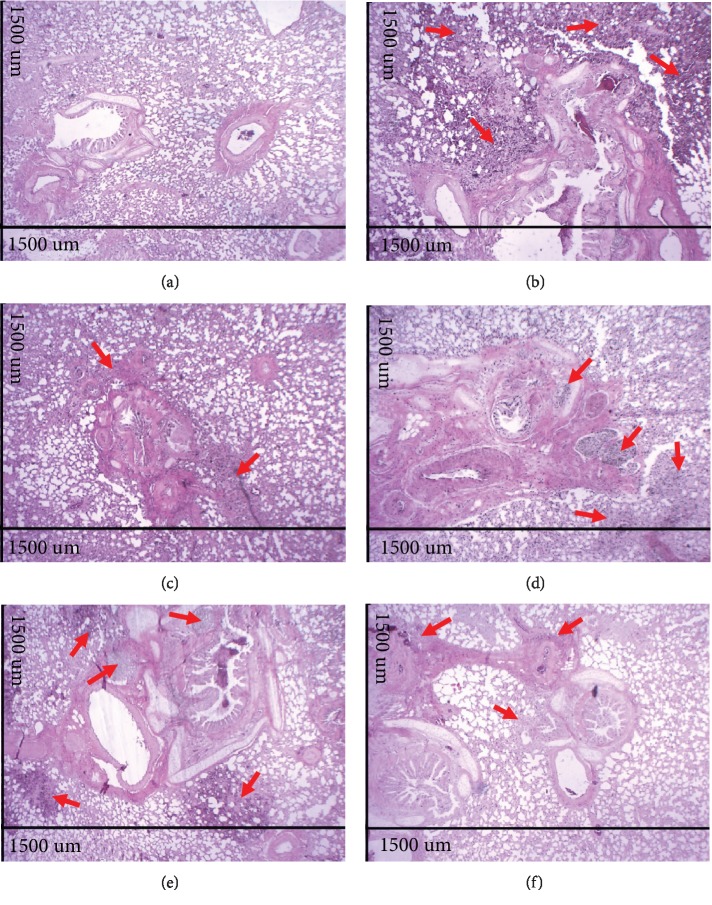
Photomicrographs of lungs of guinea pigs from Ctrl (a), Asth (b), Asth+Dexa (c), Asth+VCO1 (d), Asth+VCO2 (e), and Asth+VCO4 groups (f) showing the inflammatory infiltrate. Cellular infiltrate (red arrows). HE, A.T. ×40, 1500 *μ*m.

**Figure 3:**
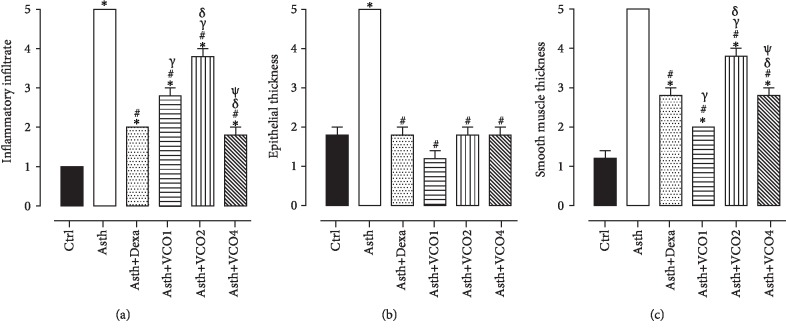
Inflammatory cell infiltrate in lung (a), epithelial hyperplasia (b), and thickness of airway smooth muscle layer (c) of guinea pigs of Ctrl, Asth, Asth+Dexa, Asth+VCO1, Asth+VCO2, and Asth+VCO4 groups. The columns and vertical bars represent the mean and S.E.M., respectively (*n* = 5). One-way ANOVA followed by Tukey's post-test: ^∗^*p* < 0.05 (Ctrl *vs.* other groups); ^#^*p* < 0.05 (Asth *vs.* other groups); ^*γ*^*p* < 0.05 (Asth+Dexa *vs.* Asth+VCO1 or Asth+VCO2); ^*δ*^*p* < 0.05 (Asth+VCO1 *vs.* Asth+VCO2 or Asth+VCO4) and ^*ψ*^*p* < 0.05 (Asth+VCO2 *vs.* Asth+VCO4).

The analysis of histological sections of the lungs in A.T. ×40 demonstrated that the Ctrl group presented normal histological appearance and preserved pulmonary histoarchitecture (Figures 2(a) and 3(a)), whereas the lungs of the Asth group showed large infiltration of inflammatory cells in the peribronchiolar and perivascular and absence of pulmonary alveoli infiltrates (Figures 2(b) and 3(a)). The treatment with dexamethasone (Figures 2(c) and 3(a)) or virgin coconut oil supplementation at doses of 1, 2, and 4 g/kg (Figures 2(d)–2(f) and 3(a)), decreases the migration from cells to the lung.

**Figure 4:**
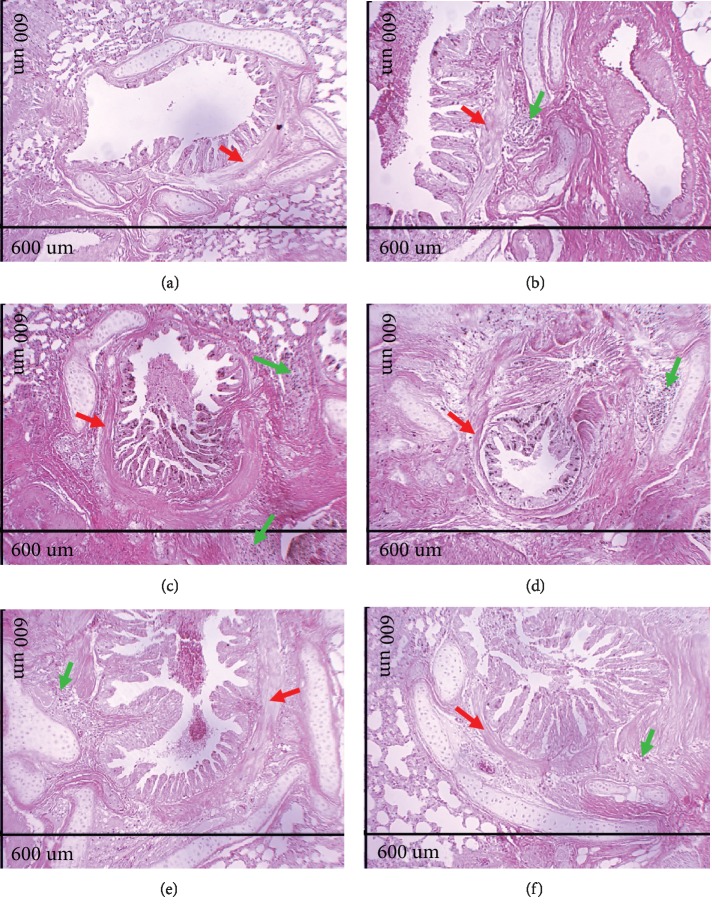
Photomicrographs of lungs of guinea pigs from Ctrl (a), Asth (b), Asth+Dexa (c), Asth+VCO1 (d), Asth+VCO2 (e), and Asth+VCO4 groups (f) showing the smooth muscle of the intrapulmonary bronchi. Intrapulmonary bronchial smooth muscle (red arrows), inflammatory infiltrate (green arrows). HE, A.T. ×100, 600 *μ*m.

The analysis of histological sections of the lungs in A.T. ×100 showed epithelial hyperplasia in the animals of the Asth group (Figures 4(b) and 3(b)) compared to the Ctrl group (Figures 4(a) and 3(b)). Treatment with dexamethasone (Figures 4(c) and 3(b)) or supplementation with virgin coconut oil at 1, 2, and 4 g/kg (Figures 4(d)–4(f) and 3(b)) reduced epithelial hyperplasia. In addition, compared to the Ctrl group (Figures 4(a) and 3(c)), the animals of the Asth group presented a greater thickness of intrapulmonary bronchial smooth muscle due to hypertrophy and/or hyperplasia (Figures 4(b) and 3(c)). Treatment with dexamethasone (Figures 4(c) and 3(c)) or with virgin coconut oil at doses of 1, 2, and 4 g/kg (Figures 4(d)–4(f) and 3(c)) reduced this thickening.

**Figure 5:**
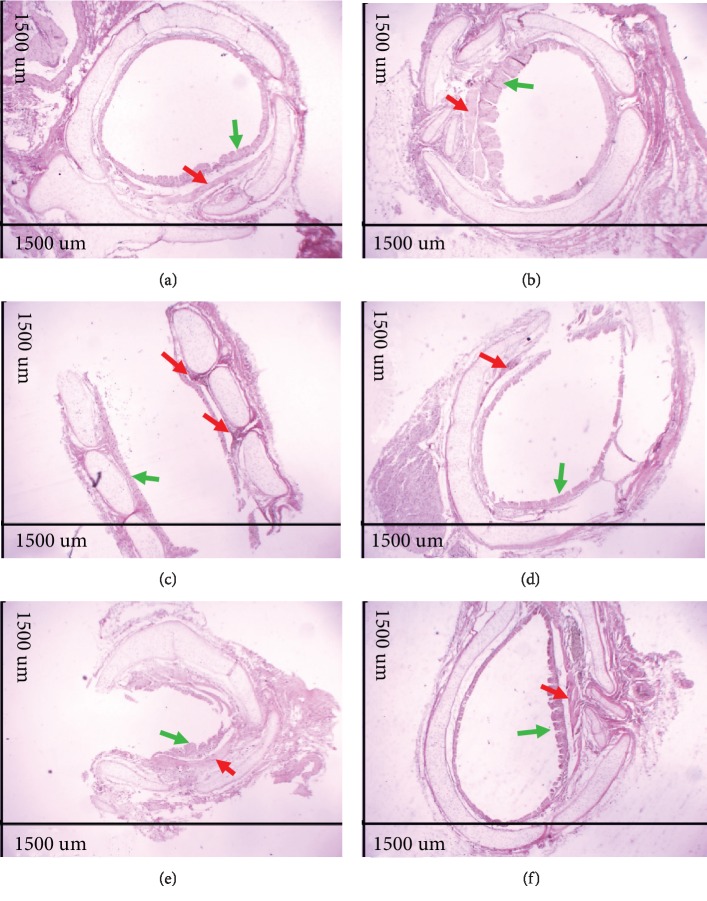
Photomicrographs of extrapulmonary bronchi of guinea pigs from Ctrl (a), Asth (b), Asth+Dexa (c), Asth+VCO1 (d), Asth+VCO2 (e), and Asth+VCO4 groups (f) showing the epithelium and smooth muscle. Smooth bronchial muscle (red arrows), epithelium and hyperplasia (green arrows). HE, A.T. ×40, 1500 *μ*m.

The analysis of histological sections of extrapulmonary bronchus in A.T. ×40 demonstrated that compared to the Ctrl group (Figures 5(a) and 3(c)), the Asth group showed bronchial smooth muscle development due to hypertrophy and/or hyperplasia (Figures 5(b) and 3(c)). Treatment with dexamethasone (Figures 5(c) and 3(c)) or supplementation with virgin coconut oil at 1, 2, and 4 g/kg (Figures 5(d)–5(f) and 3(c)) decreased the smooth muscle thickness.

### 3.2. Reactivity Measurement

#### 3.2.1. Contractile Response to Ovalbumin

The guinea pig trachea of the Ctrl group did not show contractile reactivity to OVA stimulation (*E*_max_ = 1.8 ± 0.8%, *n* = 5), differently, in the Asth group, OVA promoted contractile reactivity (*E*_max_ = 100%, *n* = 5). Neither treatment with dexamethasone (*E*_max_ = 89.1 ± 10.5%, *n* = 5) nor supplementation with virgin coconut oil at doses of 1 (*E*_max_ = 65.4 ± 8.3%, *n* = 5) and 2 g/kg (*E*_max_ = 81.6 ± 13.3%, *n* = 5) reduced the contractile reactivity of the trachea, not differing from the Asth group. Differently, supplementation with virgin coconut oil at the dose of 4 g/kg reduced the amplitude of the contraction of the trachea (*E*_max_ = 40.3 ± 2.1%, *n* = 5) in relation to the Asth group, not differing from the Ctrl group.

#### 3.2.2. Contractile Response to CCh in the Presence of Functional Epithelium

**Figure 6:**
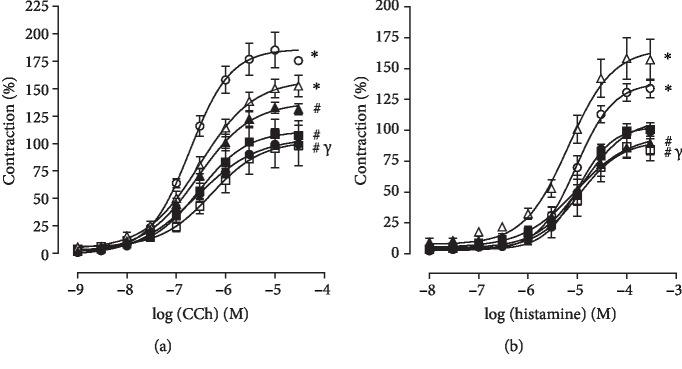
Cumulative concentration-response curves to CCh (a) and histamine (b) in guinea pigs trachea from Ctrl (black circle), Asth (white circle), Asth + Dexa (black triangle), Asth + VCO1 (white triangle), Asth + VCO2 (black square) and Asth + VCO4 groups (white square). The symbols and vertical bars represent the mean and S.E.M., respectively (*n* = 5). One-way ANOVA followed by Tukey's post-test: ^∗^*p* < 0.05 (Ctrl *vs.* other groups), ^#^*p* < 0.05 (Asth *vs.* other groups), and ^*γ*^*p* < 0.05 (Asth+VCO4 *vs.* Asth+VCO1).

In Ctrl, the trachea contracted in response to cumulative concentrations of CCh (*E*_max_ = 100%, pEC_50_ = 6.63 ± 0.10). Pulmonary inflammation, in Asth group, increased the contractile efficacy of CCh (*E*_max_ = 185.3 ± 16.1%) but did not alter its potency (pEC_50_ = 6.74 ± 0.03) compared to the Ctrl group (Figure 6(a), *n* = 5).

Treatment with dexamethasone partially prevented the increase in the contractile efficacy of CCh (*E*_max_ = 133.4 ± 4.9%) but did not change its potency (pEC_50_ = 6.59 ± 0.11), in relation to both Ctrl and Asth groups (Figure 6(a), *n* = 5).

Supplementation with virgin coconut oil at a dose of 1 g/kg did not prevent increased efficacy or potency of CCh (*E*_max_ = 153.2 ± 9.3%; pEC_50_ = 6.61 ± 0.08), compared to both Ctrl and Asth groups. However, in both Asth+VCO2 and Asth+VCO4, the increase in the contractile efficacy of CCh was completely prevented (*E*_max_ = 108.6 ± 13.7 and 98.3 ± 18.5%, respectively), with no change in potency (pEC_50_ = 6.60 ± 0.07 and 6.49 ± 0.09, respectively), in both Ctrl and Asth groups (Figures 6(a), *n* = 5).

#### 3.2.3. Contractile Response to Histamine in the Presence of Functional Epithelium

The trachea of guinea pigs from the Ctrl group contracted in response to the addition of histamine (*E*_max_ = 100%; pEC_50_ = 5.05 ± 0.04). Pulmonary inflammation, in the Asth group, increased the contractile efficacy of histamine (*E*_max_ = 132.6 ± 5.9%) but did not change its potency (pEC_50_ = 5.11 ± 0.12) (Figure 6(b), *n* = 5).

Treatment with dexamethasone completely prevented the increased contractile efficacy of histamine (*E*_max_ = 89.5 ± 9.2%) but did not change its potency (pEC_50_ = 5.21 ± 0.15), compared to both Ctrl and Asth groups (Figure 6(b), *n* = 5).

Supplementation with virgin coconut oil at doses of 1 and 2 g/kg did not prevent an increase in the contractile efficacy of histamine (*E*_max_ = 158.8 ± 16.4 and 101.5 ± 5.8%, respectively) and did not alter its potency (pEC_50_ = 5.23 ± 0.05 and 4.99 ± 0.13, respectively), compared to both Ctrl and Asth groups. In contrast, in the Asth+VCO4 group, the increased contractile efficacy of histamine was completely prevented (*E*_max_ = 87.6 ± 8.0%), but there was no change in potency (pEC_50_ = 4.99 ± 0.16), in relation to both Ctrl and Asth groups (Figure 6(b), *n* = 5).

#### 3.2.4. Role of ROS and NO Pathways in the Reactivity Measurement

**Figure 7:**
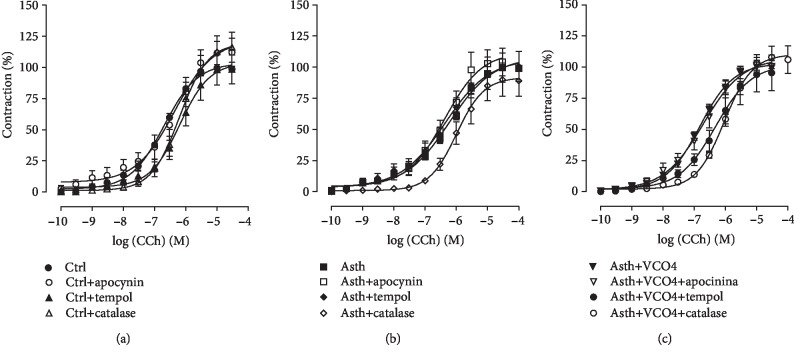
Cumulative concentration-response curves to CCh in guinea pigs' trachea from Ctrl (a), Asth (b), and Asth+VCO4 groups (c), in the absence and presence of apocynin, tempol, or catalase. The symbols and vertical bars represent the mean and S.E.M., respectively (*s*).

*(1) Cumulative Concentration-Response Curves to CCh, in the Absence and Presence of Apocynin, Tempol, or Catalase*. The cumulative concentration-response curve to CCh of the Ctrl group (*E*_max_ = 100%; pEC_50_ = 6.82 ± 0.06) was not altered in the presence of NADPH oxidase, an inhibitor of apocynin (*E*_max_ = 115.6 ± 9.4%; pEC_50_ = 6.52 ± 0.13). Differently, in the presence of tempol, a SOD mimetic, the CCh contraction curve was shifted to the right 3.2-fold (pEC_50_ = 6.30 ± 0.07), without altering the contractile efficacy of the agonist (*E*_max_ = 99, 4 ± 12.3%). Similarly, in the presence of catalase, which converts H_2_O_2_ to H_2_O and O_2_, the contractile potency of CCh was reduced by 4-fold (pEC_50_ = 6.23 ± 0.09), with no change in its efficacy (*E*_max_ = 116.6 ± 11.9%) (Figure 7(a), *n* = 5).

In the guinea pigs of the Asth group with lung inflammation, the cumulative curve to CCh (*E*_max_ = 100%; pEC_50_ = 6.35 ± 0.11) was not modified by apocynin (*E*_max_ = 105.6 ± 11.7%; pEC_50_ = 6.54 ± 0.11). Likewise, in the presence of tempol, the CCh curve was not modified (*E*_max_ = 101.0 ± 11.7%; pEC_50_ = 6.62 ± 0.27), as well as in the presence of catalase (*E*_max_ = 102.4 ± 9.1%; pEC_50_ = 5.99 ± 0.06) (Figure 7(b), *n* = 5).

In the supplementation with virgin coconut oil at the dose of 4 g/kg, it was observed that in the presence of apocynin, the CCh curve (*E*_max_ = 100%; pEC_50_ = 6.88 ± 0.07) was not altered (*E*_max_ = 102.3 ± 4.3%; pEC_50_ = 6.81 ± 0.14). In the presence of tempol, the same was observed in the Asth group, in which the potency (pEC_50_ = 6.44 ± 0.12) and the contractile efficacy of CCh (*E*_max_ = 91.9 ± 13.0%) were not altered. Differently, in the presence of catalase, the contractile potency of CCh was reduced by 6.6-fold (pEC_50_ = 6.07 ± 0.08), without the agonist's contractile efficacy being altered (*E*_max_ = 111.4 ± 8.8%) (Figure 7(c), *n* = 5).

**Figure 8:**
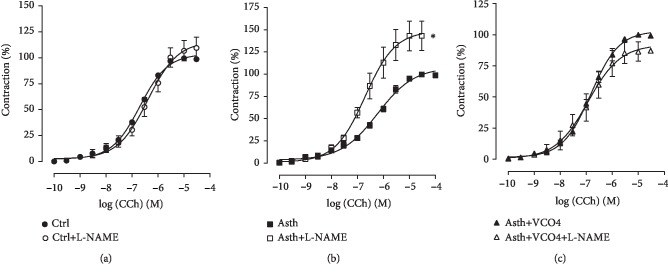
Cumulative concentration-response curves to CCh in guinea pigs' trachea from Ctrl (a), Asth (b), and Asth+VCO4 groups (c), in the absence and presence of L-NAME. The symbols and vertical bars represent the mean and S.E.M., respectively (*n* = 5). Student's *t*-test: ^∗^*p* < 0.05 (Asth+L-NAME *vs.* Asth).

*(2) Cumulative Concentration-Response Curves to CCh, in the Absence and Presence of L-NAME*. The cumulative concentration-response curve to CCh of the Ctrl group (*E*_max_ = 100%; pEC_50_ = 6.82 ± 0.06) was not altered by the NOS inhibitor L-NAME, both with respect to efficacy (*E*_max_ = 109.5 ± 10.4%) and contractile potency (pEC_50_ = 6.54 ± 0.11) (Figure 8(a), *n* = 5).

In guinea pigs of the Asth group with lung inflammation, both efficacy (*E*_max_ = 100%) and the contractile potency (pEC_50_ = 6.35 ± 0.11) of CCh were increased by L-NAME (*E*_max_ = 144.4 ± 16.0%; pEC_50_ = 6.74 ± 0.03), about 1.4- and 2.2-fold, respectively (Figure 8(b), *n* = 5).

Supplementation with virgin coconut oil at the dose of 4 g/kg promoted a similar response to that observed in the Ctrl group, and the contraction curve to CCh (*E*_max_ = 100%; pEC_50_ = 6.88 ± 0.07) was not altered by L-NAME (*E*_max_ = 87.4 ± 7.9%; pEC_50_ = 6.98 ± 0.19) (Figure 8(c), *n* = 5).

*(3) Measurement of Total Antioxidant Capacity in Plasma and Lung Tissue*. In the plasma, the animals of the Ctrl group had an antioxidant capacity value of 23.6 ± 3.6%, which did not differ from the Asth group (19.8 ± 3.2%). Dexamethasone treatment or supplementation with virgin coconut oil at all doses did not alter the plasma antioxidant capacity of guinea pigs (16.8 ± 3.0; 21.2 ± 1.5; 16.2 ± 3.4, and 17.4 ± 4.5%, respectively). In the lungs, the Ctrl group had an antioxidant capacity value of 83.0 ± 4.2%, while pulmonary inflammation of the Asth group reduced the antioxidant capacity to 59.8 ± 6.6%. Dexamethasone treatment did not alter the lung antioxidant capacity of guinea pigs with pulmonary inflammation (74.6 ± 5.0%), as well as supplementation with virgin coconut oil at the dose of 1 g/kg (77.2 ± 2.8%). However, virgin coconut oil at 2 and 4 g/kg increased the antioxidant lung capacity to 82.6 ± 5.0 and 87.8 ± 1.4%, respectively, in relation to both Ctrl and Asth groups.

**Figure 9:**
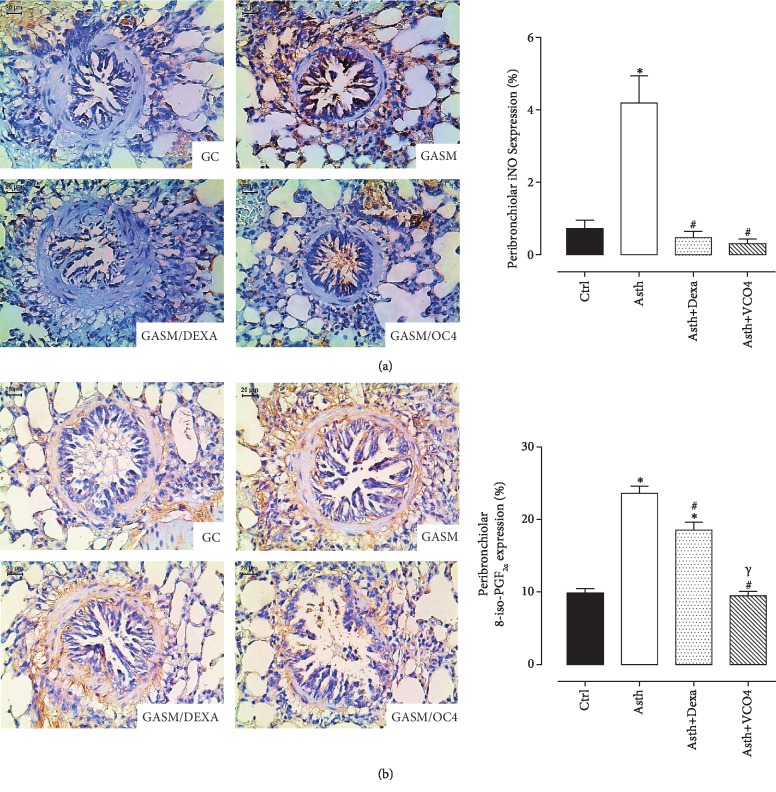
Representative photomicrographs of airway stained with immunohistochemistry to detect iNOS (a) and isoprostane (b) in Ctrl, Asth, Asth+Dexa, and Asth+VCO4 experimental groups, and the graphs representing the mean and S.E.M. of each group for iNOS and isoprostane expression in the peribronchiolar wall. ANOVA followed by Tukey post-test: ^∗^*p* < 0.05 (Ctrl *vs.* all groups), ^#^*p* < 0.05 (Asth *vs.* all groups), ^*s*^ (Asth+Dexa *vs.* Asth+VCO4).

*(4) Measurement of Peribronchiolar iNOS and 8-Iso-PGF2α Expression*. The numbers of peribronchiolar cells positive for iNOS and the volume fractions of PGF-2*α* isoprostane are shown in Figures 9(a) and 9(b) (*n* = 5). Both markers in the Asth group were increased compared to the Ctrl group. Treatment with dexamethasone attenuated these markers, as well as the supplementation with virgin coconut oil 4 g/kg.

## 4. Discussion

In this study, we have demonstrated for the first time a preventive effect of virgin coconut oil supplementation on the airway hyperactivity and oxidative damage promoted by chronic allergic lung inflammation, a model that mimic the pathologic alterations of asthma. It was shown that this prevention was obtained by increasing the pulmonary antioxidant defenses and impairing the nitric oxide-peroxynitrite-isoprostanes pathway.

Despite the large number of animal models that reproduce the characteristics of allergic asthma described in the literature, these studies have not led to new therapies for the growing number of asthmatic patients [35]. In view of this scenario, new therapeutic approaches are necessary that limit or at least make the acute attacks of asthmatic patients less frequent. In this context, it inserts virgin coconut oil, a product that has been widely used in food and industry and whose consumption has been growing mainly due to its beneficial activity on the lipid profile [36, 37] and the reduction of body fat [38, 39].

In this study, the characterization of the fatty acid profile and the chemical characterization of the virgin coconut oil were carried out in order to attest its quality. In the characterization of the fatty acid profile by gas chromatography (Supplementary data ([Supplementary-material supplementary-material-1])), the total fatty acids of the oil is mostly saturated (94.27%), followed by monounsaturated (3.99%) and polyunsaturated (1.68%). The major fatty acid in the sample was the lauric acid (66.96%), followed by myristic acid (18.90%), both medium chain, according to literature data, which point to virgin coconut oil as a “lauric oil”, due to its high content of this fatty acid [40].

In the chemical analysis, the acidity index of virgin coconut oil was relatively low, indicating the good quality of the oil, in agreement with the established by the Technical Regulation for vegetable oils and fats [41] and by the Standard for Named Vegetable Oils of FAO/UN, which establish a maximum absolute value of 4.0 mg KOH/g oil for cold pressed oils and [41] or virgin oils [42]. The peroxide index showed that the levels of peroxides in the sample of virgin coconut oil are in accordance with ANVISA [41] and FAO/UN standards [42], which determine maximum levels of 15 meq/kg of oil, for cold pressed and unrefined (or virgin) oils. Additionally, the saponification index was slightly below the range determined by the international regulatory agency [42] which establishes a value between 248 and 265 mg KOH/g oil. This can be explained by the high content of lauric acid, higher than that established by international standards, since this fatty acid is medium chain, which contributes to reduce the saponification index [42].

Virgin coconut oil has higher amounts of minor constituents like polyphenols and tocotrienols than coconut oil obtained by other methods. Additionally, it is described as antioxidant activity for these nutraceuticals from unsaponifiable fractions of the coconut oil [4, 40]. So, it is reasonable to suppose that these compounds also play a role as anti-inflammatory.

After characterization of the virgin coconut oil, we proceeded with the evaluation of its effect by food supplementation in a model of chronic allergic lung inflammation [19], based on prolonged exposure of guinea pigs to ovalbumin at increasing inhaled concentrations for four weeks at short intervals, in order to evaluate a possible modulating effect of virgin coconut oil on the pathophysiological mechanisms of asthma, both in the light of the chronic inflammatory process and bronchial hyperactivity.

Through the morphological analysis (Figures 2–3), the chronic allergic lung inflammation promotes a peribronchial infiltrate of inflammatory cells, in addition to some characteristics of the tissue remodeling, namely, hyperplasia of airway epithelial cells and thickening of the bronchial smooth muscle layer by a process of hyperplasia and/or hypertrophy. These findings are in agreement with what occurs in allergic asthma in humans, in which the remodeling process promotes the changes observed in the lungs of the guinea pigs [43–45].

Treatment with dexamethasone, as well as the supplementation with the virgin coconut oil (especially at 4 g/kg dose), reduced the migration of inflammatory cells to the pulmonary interstitium and to the peribronchial region. In addition, there was a reduction of epithelial hyperplasia, in the same proportion for both dexamethasone and three doses of the oil, and a prevention of smooth muscle thickening, being the dose of 1 g/kg more effective for this parameter (Figures 2–3). Thus, it is suggested that the effect of the oil is mainly due to an attenuation of the inflammatory process, rather than its own action on the bronchial smooth muscle and epithelial hyperplasia, which act through the release of cytokines, growth factors, and contractile mediators to promote the smooth muscle contractility characteristic of asthmatic crisis [46–48].

The effectiveness of induction of pulmonary inflammation was observed in vitro by the Schultz-Dale reaction, in which the smooth muscle of an antigenically sensitized animal contracted after reexposure to this antigen by the release of contractile mediators by the cells of the microenvironment [24; 25]. The fact that guinea pigs with pulmonary inflammation, the Asth group, presented a significant contractile response to OVA, unlike the Ctrl group, confirmed the sensitization process. This data is supported by previous studies, which showed that the trachea of asthmatic guinea pigs contracts in response to antigenic stimulation in vitro [49–51].

Dexamethasone-treated guinea pigs responded similarly to those with pulmonary inflammation upon stimulation with OVA. Although controversial, data in the literature show that corticosteroids play an important role in containing the inflammatory process, while its role on smooth muscle is still uncertain [23]. In animals supplemented with the virgin coconut oil, it was observed that, at doses of 1 and 2 g/kg, there was no change in the contractile response of the trachea, while the dose of 4 g/kg reduced the contraction by about 60% in relation to the animals with pulmonary inflammation. These data indicate that the coconut oil may partially inhibit the release of contractile factors by OVA-stimulated immune cells or negatively modulate smooth muscle contractility to reduce the bronchoconstriction observed in asthma.

In view of the primary role of smooth muscle in bronchial hyperresponsiveness, this study investigated the reactivity of guinea pig trachea in response to contractile and relaxing stimuli. An increase in the contractile reactivity of the trachea of guinea pigs with pulmonary inflammation, the Asth group, was observed in relation to nonsensitized animals, compared to CCh (Figure 6(a)) and histamine (Figure 6(b)), indicating a participation of the mechanical drug component in hypercontractility of the guinea pig trachea in pulmonary inflammation. However, it was observed that dexamethasone reversed the contractile response of the trachea to both CCh (Figure 6(a)) and histamine (Figure 6(b)), as observed by the reduction of the efficacy of these two agonists.

Supplementation with the virgin coconut oil at 1 g/kg did not prevent contractile hyperactivity of the guinea pig trachea with pulmonary inflammation against CCh (Figure 7(a)) or histamine (Figure 6(a)) but prevented CCh at doses of 2 and 4 g/kg and histamine at only the highest dose tested (Figures 6(a) and 6(b)). These results correlate with that observed for OVA stimulation, whose contraction amplitude was reduced by the dose of 4 g/kg of virgin coconut oil. These data point to a possible negative modulatory effect on the contractile mechanisms that have been altered by pulmonary inflammation, especially the expression of muscarinic and histaminergic receptors, as well as the proteins of its downstream pathways of intracellular signaling.

Asthma is characterized by an overload of reactive oxygen species, which causes oxidative stress and changes in the functions of various components of the respiratory system [52, 53]. Several works have reported the effects of ROS on the smooth muscle functioning of the airways. Hydrogen peroxide and increased oxygen levels induce contraction in the guinea pig trachea [54]. In addition to their direct effects, ROS also influence airway reactivity to contractile and relaxing agonists, highlighting the increase in the contractile response to acetylcholine and methacholine [55] and to histamine [56].

In view of this, it was investigated the contribution of ROS in CCh-mediated contraction in trachea of guinea pigs with and without pulmonary inflammation. It was observed that apocynin, a NADPH oxidase inhibitor, did not alter the cumulative curve to CCh, in guinea pig trachea preparations of Ctrl, Asth, and Asth+VCO4 (Figures 7(a)–7(c)). This data indicates that superoxide anion from NADPH oxidase plays no important role in the modulation of contractile tone of the guinea pig trachea regardless of whether or not there is a picture of allergic pulmonary inflammation and that coconut oil also does not alter this ROS formation pathway.

Differently, SOD mimetic tempol reduced the contractile potency of CCh in animals without pulmonary inflammation, but not in animals with inflamed lungs of the Asth group (Figures 7(a) and 7(b)). Similar to the Asth group, in the Asth+VCO4 group, the potency of CCh was not reduced by tempol (Figure 7(c)), therefore indicating that the superoxide anion is increased in asthmatic animals once the tempol was unable to prevent the action of this anion in the tracheal smooth muscle, but that this increase would occur through pathways independent of NADPH oxidase, such as xanthine oxidase and decoupled eNOS, also responsible for the formation of superoxide anion.

Catalase, an enzyme that converts hydrogen peroxide into water and molecular oxygen [54], reduced the contractile potency of CCh by about 4-fold in the Ctrl group; in the Asth group, the potency of CCh was not altered (Figures 8(a) and 8(b)), indicating an increase in H_2_O_2_ production triggered by the inflammatory process, reflecting the inability of catalase to reduce the levels of ROS. Interestingly, in animals with lung inflammation supplemented with the virgin coconut oil, there was a strong prevention of the formation of this free radical, since the contractile potency of CCh was reduced by 6.6-fold in the presence of catalase (Figure 7(c)). Thus, it is suggested that the coconut oil promotes its antioxidant effect in the lungs of guinea pigs with lung inflammation by reducing not the formation or clearance of superoxide anion, but the damaging effects of hydrogen peroxide on smooth muscle contractility.

Another important mediator of the contractile and relaxing reactivity of smooth muscle, in general, is NO, also produced by the tracheal and bronchial epithelium, as well as other sources such as nitrergic neurons, inflammatory cells, and smooth muscle itself [57].

The respiratory system presents the constitutive isoforms of NOS both in neurons iNANC (nNOS) and endothelium of pulmonary vessels and tracheobronchial epithelium (eNOS), involved in the regulation of airway tonus [58]. High concentrations of NO produced from iNOS have been considered to be damaging to the airways [58], since their effects are associated with the formation of the peroxynitrite free radical [56], having a strong correlation between NO levels, eosinophilia, and airway hyperresponsiveness [59, 60].

In view of this premise, it was evidenced that, while the pretreatment with the NOS inhibitor (L-NAME) did not alter the curve to CCh in the animals of the Ctrl group (Figure 8(a)), in the Asth group, both efficacy and potency of CCh were increased (Figure 8(b)). Therefore, it is assumed that, in nonasthmatic animals, there is no tonic action of NO on airway contractility, different from that observed in animals with pulmonary inflammation. It is assumed that L-NAME blocks mostly to eNOS, and little to iNOS, through which NO is predominantly formed in the Asth group, and in which eNOS is deficient.

Interestingly, in the Asth+VCO4, L-NAME did not alter the CCh contraction curve (Figure 8(c)), similar to that observed in the Ctrl group, indicating a reversibility in NO signaling promoted by the virgin coconut oil. Since the oil also had a beneficial effect on oxidative stress, it is possible to suggest a cascade effect promoted by this supplementation as the reduction in ROS and NO formation, via iNOS, with a consequent decrease lipid peroxidation.

These assumptions were confirmed by immunohistochemical data, which demonstrated that pulmonary inflammation promoted an increase in iNOS peribronchiolar expression, while dexamethasone treatment or supplementation with virgin coconut oil reduced its levels (Figure 9(a)), confirming its antioxidant protective effect for airways during asthma.

In view of the probable role of oxidative stress as a trigger for the alterations triggered by pulmonary inflammation, it was decided to evaluate the antioxidant defense in the pulmonary tissue and plasma of the guinea pigs, in order to corroborate the observed functional data. Therefore, while plasma antioxidant capacity remained unchanged in all groups, in the lung tissue, the antioxidant capacity was reduced in the Asth group, and supplementation with virgin coconut oil at doses of 2 and 4/kg increased the antioxidant capacity. However, 1 g/kg of this oil or dexamethasone treatment did not prevent this reduction.

In addition, in animals with lung inflammation, immunohistochemical staining demonstrated an increase in the levels of the 8-iso-PGF2*α*, a pro-contractile and marker of lipid peroxidation, generated as a result of nonenzymatic peroxidation of arachidonic acid in membrane phospholipids by ROS [61], followed by its reduction by dexamethasone treatment or supplementation with VCO (Figure 9(b)).

The reduction in antioxidant capacity is corroborated by studies that show similar results with other animal models of allergic asthma [62–64]. In addition, the effect observed for the coconut oil is in agreement with data from the literature, which point out its antioxidant effect as one of the benefits promoted by its supplementation [9, 40, 65–68].

In conclusion, we have shown for the first time that supplementation with virgin coconut oil emerges as promising, in light of its potential role as a functional food, in the adjuvant therapy of chronic allergic lung inflammation, especially by its actions on the inflammatory and oxidative processes of the airways that characterize asthma.

## Data Availability

No data were used to support this study.

## Supplementary Materials

Supplementary Materials Supplementary material contains representative figure of the gas chromatography of virgin coconut oil. Also includes the tables of the fatty acid content (Table 1) and the chemical index of acidity, peroxide, and saponification (Table 2) of the virgin coconut oil.
