# Supplementary material for: Virgin Coconut Oil Supplementation Prevents Airway Hyperreactivity of Guinea Pigs with Chronic Allergic Lung Inflammation by Antioxidant Mechanism
Source: Oxid Med Cell Longev. 2020 Jan 27;2020:5148503. doi: 10.1155/2020/5148503 (PMC7008286; doi:10.1155/2020/5148503)
Supplement: Supplementary Materials — Supplementary material contains representative figure of the gas chromatography of virgin coconut oil. Also includes the tables of the fatty acid content (Table 1) and the chemical index of acidity, peroxide, and saponification (Table 2) of the virgin coconut oil. [file 5148503.f1.docx]

**Supplemental Figure 1:** Gas chromatogram of virgin coconut oil. RT = retention time.

**
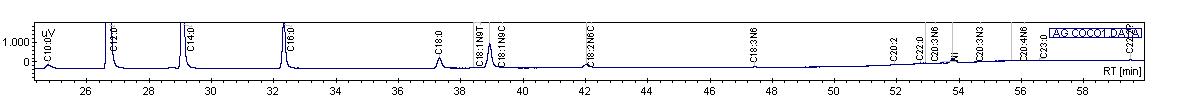

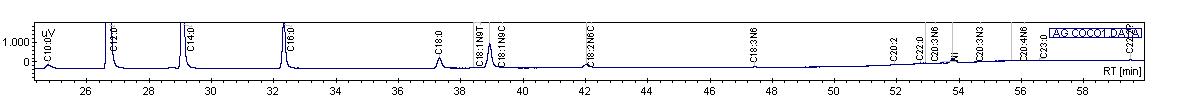

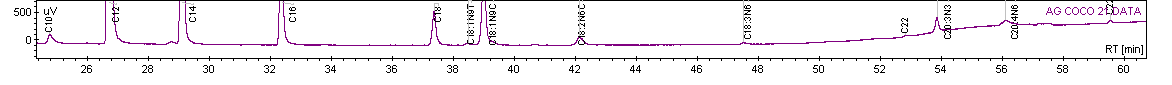

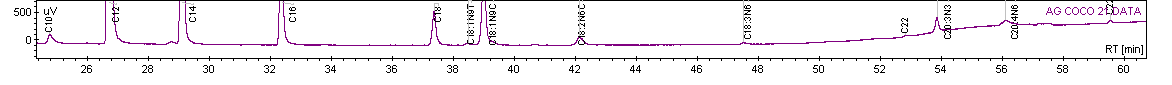
**

**Supplemental Table 1:** Profile of fatty acids present in virgin coconut oil.

| Fatty acid | | Content (%)  (mean and S.E.M) |
| --- | --- | --- |
| Usual nomenclature | **Symbology** |  |
| Capric acid | C10:0 | 0.61 ± 0.02 |
| Lauric acid | C12:0 | 66.96 ± 0.25 |
| Myristic acid | C14:0 | 18.90 ± 0.16 |
| Palmitic acid | C16:0 | 6.08 ± 0.10 |
| Stearic acid | C18:0 | 1.69 ± 0.00 |
| Behenic acid | C22:0 | 0.03 ± 0.02 |
| Total saturated fatty acids | 94.27% | |
| Elaidic acid | C18:1N9T | 0.13 ± 0.00 |
| Oleic acid | C18:1N9C | 3.86 ± 0.35 |
| Total monounsaturated fatty acids | 3.99% | |
| Linoleic acid | C18:2N6C | 0.67 ± 0.04 |
| Linolenic γ‑acid | C18:3N6 | 0.07 ± 0.02 |
| Eicosadienoic acid | C20:2N6 | 0.02 ± 0.02 |
| Di-homo‑α linolenic acid | C22:3N3 | 0.76 ± 0.17 |
| Arachidonic acid | C20:4N6 | 0.22 ± 0.14 |
| Total polyunsaturated fatty acids | 1.68% | |

**Supplemental Table 2:** Chemical analysis of virgin coconut oil.

| Parameter | index |
| --- | --- |
| Acidity | 0.28 ± 0.00 |
| Peroxide | 2.28 ± 0.16 |
| Saponification | 230.57 ± 2.07 |
